# University of Buffalo, 1846-1908

**Published:** 1908-07

**Authors:** 


A Monthly Review of Medicine and Surgery.

*EDITOR:*

WILLIAM WARREN POTTER, M. D.

All communications, whether of a literary or business nature, books for review and exchanges, should be addressed to the editor 238 DELAWARE AVE., BUFFALO, N. Y

VOL. LXIII. JULY, 1908. No. 12

### University of Buffalo, 1846-1908

THE sixty-second annual commencement of the department of medicine, the twenty-first annual commencement of the department of pharmacy, the twentieth annual commencement of the department of law and the fifteenth annual commencement of the department of dentistry of the University of Buffalo took place at the Teck theater, Friday, May 29, 1908, at 11 A. M. The preliminary announcement of the exercises during commencement week was published in the June issue of the JOURNAL. The magazine, however, went to press too early to give the full proceedings, hence we take pleasure in publishing them in this edition.

ALUMNI ASSOCIATION.

The Alumni Association of the medical department held its thirty-third annual meeting during Tuesday, Wednesday and Thursday, May 26, 27, and 28, 1908. The program for Tuesday evening, May 26, was as follows: at 8.00 P. M., the classes of '48, '58, '68, '78, '83, '88 and '98 met in the college building for organisation and registration; at 8.30 P. M., the association transacted routine business and elected officers for the ensuing year; at 9.00 P. M., vice-chancellor Charles P. Norton welcomed the classes in reunion and the association, and told more about Buffalo's Greater University. President Allen A. Jones, '89, delivered the annual address; Dr. Earl P. Lothrop, '94, described the Emanuel Church Movement of Boston; Dr. Frederick C. Busch, '97, Professor of Physiology, reviewed recent discoveries in physiology. At 10.30 P. M., an informal reception by the Faculty of the department was held. At 11 P. M., a smoker was held in the Library of the College at which light refreshments were served.

The officers elected for the ensuing year were: president, Albert T. Lytle, '93; first vice-president, Fitch H. Van Orsdale, '91, Belmont; second vice-president, Charles L. Preisch, '98, Lockport; third vice-president, W. H. Knickerbocker, '97, Geneva; fourth vice-president, Alice Bennett, '90; fifth vice-president, Ross G. Loop, '97, Elmira; treasurer, Herman K. DeGroat, '97, and Franklin W. Barrows, '93, secretary. The executive committee will consist of Marshall Clinton, '95; Fred G. Parmenter, '03; Lawrence Hendee, '97, and Albert T. Lytle and Eli H. Long, as ex-officio members.

Beginning Wednesday morning the museum of pathology was established under the supervision of Drs. Williams, Gibson and Busch, in which specimens, preparations and photographs were exhibited. During Wednesday and Thursday clinics were held at the several hospitals in accordance with a carefully prepared schedule. Wednesday evening, May 27, was set aside as a fraternity night. The several secret societies of the under classmen, the "I. C. I.," the "A. O. D.," the "O. U. P.," and the "N. S. N.," each prepared a program and issued invitations to the visiting alumni. Thursday evening, May 28, was called free lance night, during which class dinners, smokers and theater parties were given.

UNIVERSITY DAY.

The annual commencements of the several departments of medicine, pharmacy, law and dentistry were held at the Teck theater, Friday, May 29, 1908, commencing at 11 o'clock in the morning. The theater was filled with friends of the graduates and gay with the university colors and masses of flowers.

The program was made up of orchestral music, "Alma Mater" sung by the student body, prayer by Rev. Richard Earle Locke, pastor of Calvary Presbyterian Church, the conferring of degrees and announcement of honors, the address to the graduates by Rev. Samuel Van Vranken Holmes, D. D., pastor of the Westminister Church, and the benediction by Rev. Mr. Locke.

The vice-chancellor, Charles P. Norton, Esq., presided and conferred degrees upon one hundred and twenty-one graduates, thirty-seven of whom received the degree of doctor of medicine, 30 were given the degree of LL. B., 30 became bachelors of pharmacy and 24 received the degree of doctor of dental surgery. In addition to degrees awarded to younger men, the degree of master of laws was awarded to six.

In the department of medicine, in the illness of Dr. Matthew D. Mann, dean of the college, the hippocratic oath was administered by Dr. Charles Cary, who also announced the class honors. The presentation of degrees was by Dr. Eli H. Long. These were the honor men: first, Clayton Halsey Snover; second, Hugh B.

Deegan; third, John Henry Evans; fourth, Joseph Albert Gregory.

The graduates in medicine were:

Arnold, Douglas Perkins, 845 Richmond Ave., Buffalo; Borowiak, Stanislaus Nicholas, 69 Rother Ave., Buffalo; Brennan, Joseph Patrick, 59 Cleveland Ave., Buffalo; Brumberg, David, 31 Walnut St., Buffalo; Cott, Chester Charles, 330 Depew Ave., Buffalo; Deegan, Hugh B., Dansville, N. Y.; Ende, Edward Henry, 535 Clinton St., Buffalo; Eschelman, Karl Ferdinand, D. D. S., 421 Franklin St., Buffalo; Evans, John Henry, Machias, N. Y.; Gianfranceschi, Joseph Shem, 338 Seneca St., Buffalo; Gow, Edward Cochrane, Schuylerville, N. Y.; Greene, Christiana Marion, 152 Bird Ave., Buffalo; Gregory, Joseph Albert, 149 Riley St., Buffalo; Gunn, Lee, Hamburg, N. Y.; Haenszel, Allen Lee, 316 Landon St., Buffalo; Hahl, Arthur Otto, 82 Monroe St., Buffalo; Hummell, Harry Charles, Lancaster, N. Y.; Jacobs, William F., 89 Riley St., Buffalo; Lawler, Arthur V., 600 W. Clinton St., Elmira N. Y.; McKee, Otto Sexsmith, Ph. B., Lockport, N. Y.; Maichle, Robert Jacob, Cohocton, N. Y.; Reusch, George F., Belfast, N. Y.; Reynolds, George Goff, 305 Desmond St., Sayre, Pa.; Richman, Raynauld D., Morton, N. Y.; Roe, Jesse Nathaniel, 97 High St., Buffalo; Ryan, John F., 83 N. Division St., Buffalo; Seyse, Arthur L., Strykersville, N. Y.; Snover, Clayton Halsey, Steamburg, N. Y.; Stesel, George Augustus, 72 Goodrich St., Buffalo; Stowe, John Gurney, 758 Elmwood Ave., Buffalo; Terrasse, Frederick, 140 Locust St., Buffalo; Valanti, Frank A., 286 Front Ave., Buffalo; Van Campen, Benjamin, A. B., 122 N. First St., Olean, N. Y.; Waters, LaVerne Francis, Medina, N. Y.; Williamson, Claude Cobb, Clifton Springs, N. Y.; Wright, Frederick L., 374 Summer St., Buffalo; Wurtz, Walter J. M., 91 Krettner St., Buffalo.

Prizes were also awarded in the departments of pharmacy, law and dentistry. Upon conclusion of the exercises at the theater the faculty of the medical department received the graduates, their friends and the alumni at the University club where luncheon was served.

---

FRATERNITY Night May 27, was observed with banquets and reunions by all the fraternities connected with the University of Buffalo. The I. C. I. Chapter of the Nu Sigma Nu Fraternity met in the Chapter House, 97 High street, where an excellent program of speeches was presented. Dr. Charles G. Stockton presided, and among the other speakers were Allen A. Jones, Albert T. Lytle, James W. Putnam, Roswell Park, Herbert U. Williams, Ernest Wende, Walter D. Greene and William C. Phelps.

Two reunions of medical classes of the university were held May 28, 1908, that of the class of '88 at the University Club and that of the class of '98 at the Hotel Statler. At the former, attended by 12 members, Dr. George H. McMichael, president, acted as toastmaster, the following officers being elected: president, Henry C. Buswell; vice-presidents, Charles Sumner Jones, and H. L. Hunt, of Orchard Park; secretary, C. Clark Ernest; executive committee, W. Henry Woodbury, Edwin L. Wood of Dansville, John J. Finerty, E. N. Pfohl and George H. McMichael.

President T. H. McKee presided over the banquet of the class of '98, for which 52 covers were laid. Four women physicians were present: W. M. Tompkins, New York; Edith W. Stewart, Hume, N. Y.; Loretta Wooden-Turner, Rochester, and Annie May Cheny-Spofford, Batavia, N. Y.

---

Henry Phipps, of Pittsburg and New York, just prior to sailing for Europe June 13, 1908, according to an associated press despatch, arranged for a gift to Johns Hopkins Hospital and University for the founding of a psychiatric clinic, on the lines of well known similar institutions in Europe. It will be the first of its kind with adequate equipment and support in connection with a large hospital and university in this country.

The funds provide for the construction of a four-story hospital building on the Hopkins Hospital grounds to accommodate sixty patients, together with rooms for private patients, modern apparatus for use in the treatment of patients, and laboratories for the scientific investigation of mental abnormalities by pathological, chemical and psychological methods. In addition, Mr. Phipps will provide for the maintenance of a medical and nursing staff of a high order, including salaries for a professor of psychiatry and assistants and other expenses for a period of ten years. The total amount of the gift is withheld, in accordance with the wishes of Mr. Phipps, but it is understood that it will considerably exceed $500,000. Mr. Phipps makes the gift upon his own initiative.

Dr. Adolph Meyer, professor of psychiatry in the medical department of Cornell University, New York, has been elected to the directorship of this recently founded psychiatric clinic and to the professorship of psychiatry. Dr. Meyer has accepted.

---

Women medical students are winning laurels this year. Miss Jeannette Miller, of Massillon, Ohio, has just been graduated with the highest honors of her class from the Cleveland College of Physicians and Surgeons. The day after her graduation Dr. Miller took charge of the Maternity Dispensary Hospital in Cleveland. She will remain there three months and then engage in private practice.

For the first time in several years a woman graduate delivered the valedictory address for the graduating class of the Eclectic College of New York. Her name is Miss Stella Schaffer, and she is also the winner of the electrotherapeutic prize—an electric battery.

A woman from far off Cavite, in the Philippines, Miss Olive Salamanca, carried off the Agnes B. Robinson-Mesner prize in anatomy, given on competitive examination to a student of the second year at the Philadelphia Woman's Medical College. Miss Ethel Das, who comes from Ferozepore, a town in the foothills of the Himalayas, near Lapore, is another member of Miss Salamanca's class. Both of these young women will go back to their native countries to practise medicine.

---

A BULLETIN of the Connecticut State Board of Health recently issued calls attention to the dangerous character of the ordinary house fly, justly accusing that busy insect of all manner of offences against the public health. The secretary of the board says: "We have had occasion frequently to comment in the columns of this bulletin on the cause and prevention of typhoid fever, and, so long as this disease continues to be a living issue among us, we shall continue to do so. Water, milk, oysters and flies have at different times been spoken of as means of spreading this disease. It is a significant fact that typhoid is most prevalent at the season of the year when flies are the most numerous. These insects breed by preference in stable manure, but, when this is not readily accessible, will breed also in garbage and other filth. With cleaner streets, the better care of stables, back yards, markets and cleaner garbage pails, the breeding places of flies will be limited and their agency in carrying the typhoid and other bacilli to the food of human beings will be less marked. Meanwhile, the screening of our houses is not a luxury, but a necessity."

---

AMERICAN army surgeons have had such an honorable and conspicuous share in proving that mosquitoes disseminate disease that there is special propriety in the campaign against these insects which the War Department has just organised. In all probability civilians as well as soldiers will be benefited by the abatement at military posts of what is both a nuisance and a menace to health.

---

DR. Andrew S. Draper, State Commissioner of Education, was the guest of honor at a dinner tendered by the regents of the University of the State of New York and the officers of the state education department, served at the Hotel Ten Eyck, Albany, on the evening of June 22, 1908. The event commemorated the sixtieth birthday anniversary of the distinguished educator. Vicechancellor McKelway presided and addresses were made by regents McKelway, Sexton, Philbin and Lauterbach and Dr. Howard J. Rogers, first assistant commissioner of education. Many congratulatory letters and telegrams were received.

Commenting on Dr. Draper's conspicuous services to the state the *Buffalo Evening News* in its issue of June 22, 1908, said.

When the school law of the State was reconstructed, four years ago, Dr. Draper was called to his early home to become the head of the new system. It is needless to speak of his brilliant success in that position. No other man in the United States, or in any other country for that matter, speaks with higher authority than Dr. Draper on the subject that enlists his talents and his heart alike. No one is doing better work than he in the cause of popular education. No one surpasses him in excellence of exposition of themes connected with it or shows more convincingly the vital relation between education and our national institutions. It is in the highest degree fitting that his life and work be celebrated in the manner proposed.

---

THE regents of the University of the state of New York held a special meeting in their rooms in the capitol at Albany, June 23, 1908, and appointed the following named members of the state board of medical examiners: Frank W. Adriance, Elmira; Floyd S. Farnsworth, Plattsburg; and Ralph H. Williams, Rochester. These are all reappointments to fill vacancies created by expiration of their own terms and these members are to hold office for three years from August 1, 1908.

---

"WE could duplicate our navy, fortify our coasts, deepen our navigable waterways, pay the last dollar on the Panama canal and wipe out our national debt in ten years by stopping the waste of a single one of our natural resources, and a natural resource, too, that does not seem to have been mentioned at the conference of governors recently held at Washington", declared Dr. Charles A. L. Reed, of Ohio in an address recently before the alumni of the Northwestern University. "This resource is the productive energy of the people without which the soil, the mines, the forests and the waterways would be as worthless as the sandy stretches of the Sahara. The waste of this energy through preventable diseases alone amounts to a billion dollars annually." Figures were given from the latest government statistics, to prove this assertion. The saving, it was claimed, could be effected through the operations of a National Department of Public Health, the creation of which is now being demanded by the people. Various state platforms have already declared for such a department. "We now ask," said Dr. Reed, "in the name and in the interest of all the people that the great political parties make similar pledges in their national platforms this year and that they redeem those pledges when the Congress shall again convene."

